# Application of a Novel and Improved VGG-19 Network in the Detection of Workers Wearing Masks

**DOI:** 10.1088/1742-6596/1518/1/012041

**Published:** 2020-04

**Authors:** Jian Xiao, Jia Wang, Shaozhong Cao, Bilong Li

**Affiliations:** 1 No.l (band-2) Xinghua Street, Daxing District, Beijing, Beijing Institute of Graphic Communication xiaojianece@163.com

## Abstract

In order to work and travel safely during the outbreak of COVID-19, a method of security detection based on deep learning is proposed by using machine vision instead of manual monitoring. To detect the illegal behaviors of workers without masks in workplaces and densely populated areas, an improved convolutional neural network VGG-19 algorithm is proposed under the framework of tensorflow, and more than 3000 images are collected for model training and testing. Using VGG-19 network model, three FC layers are optimized into one flat layer and two FC layers with reduced parameters. The softmax classification layer of the original model is replaced by a 2-label softmax classifier. The experimental results show that the precision of the model is 97.62% and the recall is 96.31%. The precision of identifying the workers without masks is 96.82%, the recall is 94.07%, and the data set provided has a high precision. For the future social health and safety to provide favorable test data.

## 1.Introduction
In printing factories, there are a large number of toxic chemicals such as inks and pigments, and the dust formed by these substances may damage workers' respiratory health. Therefore, proper wearing of masks is the focus of safety production management [1-2]. In order to curb the phenomenon of violation in printing factories, it is necessary to use modern machine vision means to achieve real-time, accurate and predictable mask wearing detection.

With the development of basic science such as neurophysics, artificial intelligence and visual computing theory, as well as the gradual improvement of computer hardware computing power, machine vision has ushered in a broader application prospect [3]. Relevant scholars have done a lot of research on image recognition technology in computer vision to prevent accidents. Seo et al. proposed the use of computer vision technology for object detection, object tracking and action recognition of the three aspects of unsafe environment and unsafe behavior identification, for the factory safety detection provided a theoretical and technical basis [4]. In order to realize the supervision of machine vision, this paper further analyzes the wearing situation of workers' masks by combining video human body recognition technology, and thus proposes an improved algorithm of convolutional neural network VGG-19 using deep learning to achieve this goal.

## 2. Method
Based on the TensorFlow framework, this paper identifies the staff wearing masks and those without masks in the images of the printing plant. Firstly, a certain number of mask images are collected as the training and test set of the model. Since the image pixels collected are too large, which increases the

Content from this work may be used under the terms of the Creative Commons Attribution 3.0 licence. Any further distribution of this work must maintain attribution to the author(s) and the title of the work, journal citation and DOI.
Published under licence by IOP Publishing Ltd memory processing burden of the computer, the images need to be preprocessed accordingly to meet the requirements of the TensorFlow framework for image recognition. Then, VGG-19 DCNN was constructed, and the model parameters were fine-tuned according to the model training and test results. VGG-19 Net extracted the low-level and high-level features of images layer by layer, and finally realized image classification, so as to achieve the recognition accuracy requirements. The main identification process in this paper is divided into data collection, data preprocessing, model training, model test and model test.

### *2.1 VGG-19 Network*

VGG CNN has six main structures, each of which is mainly composed of multiple connected convolutional layers and full-connected layers. The size of the convolutional kernel is 3*3, and the input size is 224*224*3. The number of layers is generally concentrated at 16~19 [5]. The VGG-19 model structure is shown in Figure 1 [6].

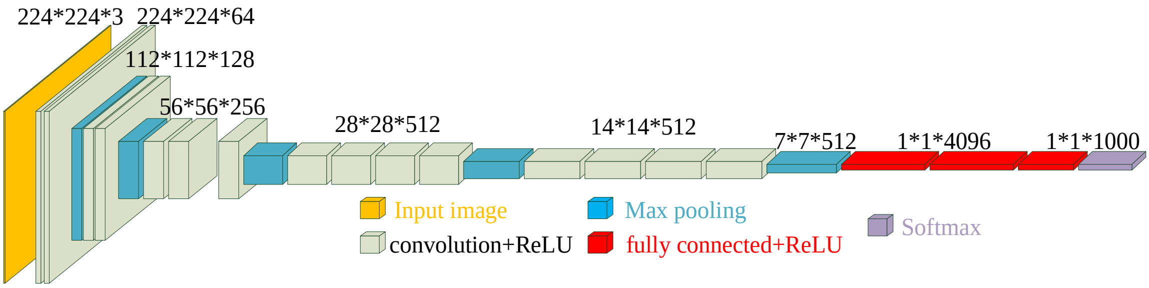

**Figure 1.** VGG-19 network model

VGG-19 CNN is used as a pre-processing model. Compared with traditional convolutional neural networks, it has been improved in network depth. It uses an alternating structure of multiple convolutional layers and non-linear activation layers, which is better than a single convolution The layer structure can better extract image features, use Maxpooling for downsampling, and modify the linear unit (ReLU) as the activation function, that is, select the largest value in the image area as the pooled value of the area. The downsampling layer is mainly used to improve the anti-distortion ability of the network to the image, while retaining the main features of the sample and reducing the number of parameters [7]. The expression for the downsampling layer is (1). Among them, $down\left(\chi_j^{(n-1)}\right)$ is the maximum pooling sampling function, $\tau_j^n$ is the coefficient corresponding to the j-th feature map of the n-th layer, and $f\left(\tau_j^n down\left(\chi_j^{(n-1)}\right)+b_j^{(n)}\right)$ is the ReLU activation function.

$$\chi_{p_j}^{(n)} = f\left(\tau_j^n down\left(\chi_j^{(n-1)}\right)+b_j^{(n)}\right) \tag{1}$$

### *2.2 Mask wearing detection using improved VGG-19 network model*

Because of the variety of mask colors and the state in the scene, and the changes in the working environment light, traditional detection methods are difficult to address the above issues. This paper uses a deep learning convolutional neural network model-based target detection and positioning method, and proposes an improved VGG model. The trained VGG-19 network is used as a pre-trained model of this model. By fine-tuning the transfer learning method, the parameters of the pre-trained model optimize the model parameters of the convolution layer and solve the classification problem of wearing masks. The parameters in VGG-19 are concentrated in 3 FC layers. The parameters of the network were originally designed for 1000 classification [8], but this article only focuses on the classification of 2 categories (whether wearing masks). Therefore, it is proposed to replace the three fully connected layers of VGG-19 with one Flatten layer and two fully connected layers. Since the convolution layer cannot be directly connected to the Dense fully connected layer, a Flatten layer is added. The improved model training framework mainly uses the fine-tuning transfer learning to transfer the VGG-19 pretrained model parameters to the convolution layer, pooling layer and fully connected layer of the mask wearing detection model, and replaces the original with a 2-label Softmax classifier Softmax classification layer, sparse features through Dropout, Max pooling, and fit a detection model with good accuracy, as shown in Figure 2.

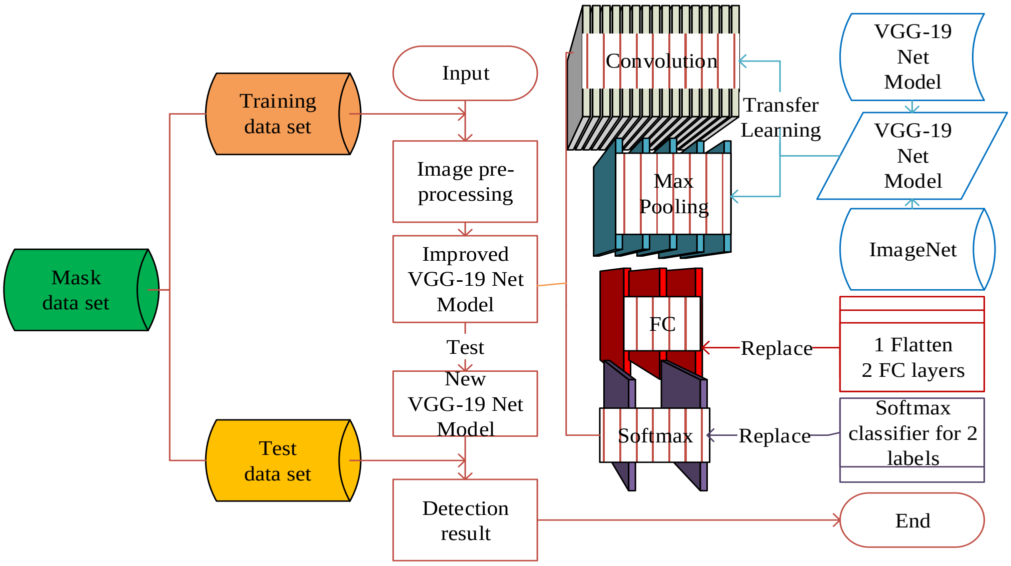

**Figure 2.** Training frame of mask wearing detection model

The main operation process is as follows:

(1) Enter a sample picture of wearing a mask. The pictures are extracted from the library of positive and negative images of mask wearing as a training sample set for input.

(2) Pre-processing. In order to improve the training efficiency, the input image is standardized to a resolution of 300 * 300.

(3) Construct new and improved models. using the VGG-19 Net model, the 3 FC layers are optimized as 1 Flatten layer and 2 FC layers with reduced parameters. Replace the Softmax classification layer of the original model with a 2-label Softmax classifier.

(4) Micro-transfer learning. Using the parameters of the 16 convolutional layers and pooling layers of the VGG pre-trained model, the parameters of the detection model were optimized by transfer learning.

(5) Model training. To train and optimize the parameters of 2 FC layers and Softmax layers, it is necessary to freeze the parameters of 16 convolutional layers and their pooling layers and initialize the model parameters using a random method, set the momentum parameters, the learning rate, and the accuracy standard to iterate.

(6) Model testing. Extract the remaining positive and negative sample pictures from the data set as the test sample set for model testing, and calculate the precision and recall.

*2.3 Model evaluation criteria*

The evaluation of the mask wearing detection network model can be evaluated from the effect and reliability. These two indicators are usually the precision and the time taken for testing, while the former includes three indicators: precision, recall rate and error rate. The test time refers to the time it takes for the algorithm to test one picture. Since the security detection requirements meet the real-time nature, the short test time of the algorithm has important practical significance. Among them, precision is defined as (2), and the recall is defined as (3). The Error rate is defined as (4).

$$P = \frac{TP}{TP+FP} \tag{2}$$

$$recall = \frac{TP}{TP+FN} = \frac{TP}{P} \tag{3}$$

$$Error\ rate = \frac{FP+FN}{TP+TN+FP+FN} = \frac{FN}{TP+FN} = \frac{FN}{P} = 1 - recall \quad (4)$$

Among them, TP is the number of positive samples determined by the model to be positive, TN is the number of negative samples determined by the model to be actually negative, and FP is the number of positive samples determined by the model However, the actual number of negative samples is FN, which is the number of negative samples determined by the model but actually positive samples, as shown in Tables 1 [9].

**Table 1.** TP, TN, FP and FN parameter definitions

| | Positive | Negative |
|---|---|---|
| True | TP | TN |
| False | FP | FN |

## 3 Results and discussion

The novel model was trained and tested. One was equipped with an Intel Core i7-6850K 3.60 GHz 12-core processor, one NVIDIA GeForce GTX 1080 Ti GPU 27604MB. On the deep learning workstation platform, this platform is installed with Windows 10 operating system, python3.6 / 3.7 and TensorFlow 2.0 framework. It can be used to build a model framework for mask wearing recognition based on VGG-19 Net improvement and training and testing through Python language compilation. In this paper, the data of the web crawler is used as training samples. Repetitive and non-standard data are removed manually, and preprocessing operations such as rotation, scaling, and translation are performed on the image. The final number of training samples is 3261. The data set was divided into two positive and negative samples. The positive samples were 1435 faces with masks (Mask), and the negative samples were 1826 faces without masks (Un-mask). After the standard positive and negative data sets are established, the mask area of all pictures in the sample set is manually labeled with the LabelImg tool, and the information such as the coordinates of the rectangular frame and the corresponding picture file name are saved as an xml file. 20% of the training sample data is provided as the validation set, of which the number of training set samples is 2609 and the number of validation set samples is 652.

### *3.1 Training experiment*

In order to avoid the impact of sample imbalance on the model as much as possible, the collected 3261 images are used as the training set of the model, and the image is pre-processed by pixel scaling. Stickiness and generalization ability to prevent overfitting. Set the training set and test set in two folders according to the ratio of 8: 2. Among them, there are 1148 training sets for positive samples and 287 test sets. 1461 training sets for negative samples and 365 tests set.

The threshold of target detection in deep learning refers to a threshold value for the algorithm to distinguish positive and negative samples. The model finally outputs the target area score through the Softmax layer to achieve classification [10]. For example, the model score of 90% means that the probability that the VGG-19 algorithm calculation box selects the target as a true label is 90%. During the threshold setting process, the algorithm tends to accept the object frame area closer to the true label for the setting of the high threshold value, discarding the object frame area with lower scores, causing the model to generate high precision and low recall. Instead, the low threshold Biased to accept low-scoring object frame areas, resulting in high recall and low precision. Due to the highly complex background of the printing industry site, it poses a great challenge for workers to wear masks. Therefore, it is necessary to consider the "equilibrium state" between the precision and the recall rate. After a large number of experiments, the threshold is set to 0.5 to ensure that the model has a high precision and recall. During training, in order to avoid the loss of data diversification caused by too fast gradient descent, the number of iterations of the network model is set to 20, and the number of batches per iteration is 108, that is, the number of iterations is 2160, and the learning rate is 0.001. Derive the loss value and plot the iterative loss value curve of the network training model. Figure 3 is a function trend graph of the loss value and the number of iterations trained according to the above steps. The abscissa is the number of iterations and the ordinate is the loss value. It can be seen that the loss value decreases with the number of iterations within a certain range. The image training time takes 3.5h and eventually the loss value tends to a stable minimum value of 0.0297 while avoiding other conditions from overfitting.

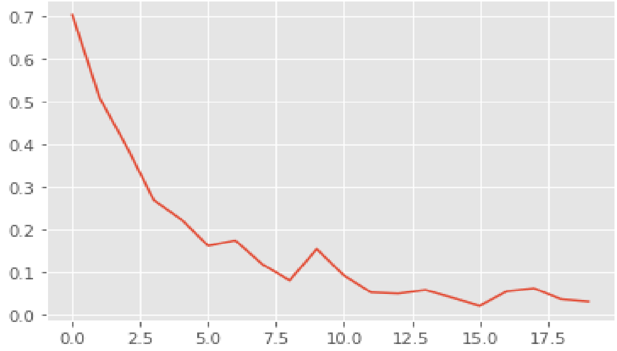

**Figure 3.** Relationship between loss value and number of iterations

*3.2 Testing and evaluation*

The test of the improved recognition of VGG-19 network mask wearing includes two parts of machine vision and human vision, that is, the VGG-19 network model recognition of face mask wearing and the actual recognition of human eyes. Place 652 images in the test folder and call the trained and improved VGG-19 network model for recognition. At the same time, the TP, FP, TN, and FN parameters of the model are identified and counted by human observation. According to the method described above, the effectiveness of the undetected and improved VGG-19 Net identification worker safety detection was evaluated. Calculate according to the calculation formulas of the precision, recall rate, and error rate above. The recognition results are shown in Table 2.

**Table 2.** Recognition effect of unimproved VGG-19 network

| | TP | FP | TN | FN | precision | recall | Error rate |
|---|---|---|---|---|---|---|---|
| Mask | 287 | 44 | 365 | 51 | 86.71% | 84.91% | 15.09% |
| Un-mask | 365 | 51 | 287 | 61 | 87.74% | 85.68% | 14.32% |

The improved VGG-19 Net has a very good effect compared to mask wearing recognition. As shown in Table 3, the average precision of identifying wearing a mask is 97.62%, and the recall rate is 96.19%. The average precision of identifying wearing a mask is 96.74%, and the recall rate is 93.89%. The time and improvement of testing a picture the front is almost unchanged for 0.7s, and its test chart is shown in Figure 4. (a) is the face selected by the blue frame when the mask is not worn, and (b) is selected by the green frame when the mask is worn.

**Table 3.** Recognition effect of improved VGG-19 network

| | TP | FP | TN | FN | precision | recall | Error rate |
|---|---|---|---|---|---|---|---|
| Mask | 287 | 7 | 365 | 11 | 97.62% | 96.31% | 3.69% |
| Un-mask | 365 | 12 | 287 | 23 | 96.82% | 94.07% | 5.93% |

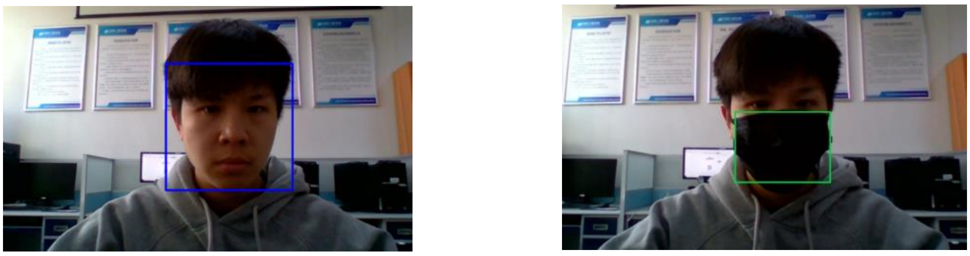

(a) (b)

**Figure 4.** Test legend

In summary, the precision of the improved network model for detecting whether or not to wear a mask has increased by 10.91% and 9.08%, respectively, and its recall rate has increased by 11.4% and 8.39%, respectively. Correspondingly, the error rates were reduced by 11.4% and 8.39%, respectively. It can be known that this improvement effect is relatively significant. And the parameters have been greatly reduced, so the improved vgg-19 mask wearing detection network model has a good potential for workers' safety detection, and provides a favorable guarantee for the chemical industry factory's safety pre inspection.

## 4 Conclusion

This paper uses VGG-19 Net to replace the original 3 FC layers with 1 Flatten layer and 2 FC layers, and replaces the original Softmax classifier of VGG-19 with 2 labeled Softmax classification layers. Training and testing tests on masked people (Mask) and unmasked workers (Un-mask) concluded that:

1) Within a certain range of network model training times, the greater the number of iterations, the higher the model's recognition detection rate, and beyond a certain range, the network model's recognition precision will not continue to increase, and it will even decrease to a certain extent The range of the number of training steps for the subsequent network model does not exceed 1944.

2) Tests on the detection of masks worn by workers show that the improved algorithm can achieve a good recognition effect in the detection of mask wearing, which verifies the effectiveness and feasibility of the algorithm's practical application.

3) The research model in this paper is improved in the FC layer of VGG-19 Net. Later research can increase the optimization of the convolutional layer and pooling layer of the network model, further optimize the network, and expand in complex scenarios Detection and identification of medium safety equipment.

## Acknowledgments

This paper is supported by the scientific research program of Beijing Municipal Education Commission. The project name is "Research on the intelligent recognition system of printing roller surface defects based on machine vision" (No. KZ202010015021).
